# Circularly symmetric nanopores in 3D femtosecond laser nanolithography with burst control and the role of energy dose

**DOI:** 10.1515/nanoph-2022-0665

**Published:** 2023-01-11

**Authors:** Franzette Paz-Buclatin, Marcos Esquivel-González, Alfredo Casasnovas-Melián, Omar de Varona, Carlos Cairós, Juan Manuel Trujillo-Sevilla, Kei Kamada, Akira Yoshikawa, Jose Manuel Rodríguez-Ramos, Leopoldo Luis Martin, Airan Ródenas

**Affiliations:** Department of Physics, Universidad de La Laguna, La Laguna, Spain; Department of Physics, Universidad de La Laguna, Avda. Astrofísico Francisco Sáncehez, S/N, Facultad de ciencias, La Laguna, Santa Cruz de Tenerife 38200, Spain; Departamento de Ciencias Medicas Basicas, Universidad de La Laguna, La Laguna, Spain; Wooptix SL, San Cristóbal de La Laguna, Spain; Tohoku University Institute for Materials Research, Sendai, Miyagi, Japan

**Keywords:** 3D laser writing, 3D nanofabrication, 3D nanolithography, 3D nanophotonics, laser nanostructuring, nanofabrication

## Abstract

The fabrication of three-dimensional (3D) nanostructures within optical materials is currently a highly sought-after capability. Achieving nanoscale structuring of media within its inner volume in 3D and with free design flexibility, high accuracy and precision is a development yet to be demonstrated. In this work, a 3D laser nanolithography technique is developed which allows producing mm-long hollow nanopores inside solid-state laser crystals and with a high degree of control of pore cross-sectional aspect ratio and size. We report an in-depth study on the formation of pores both within the non-thermal regime at which temperature is fast dissipated after each laser pulse, and for a thermally controlled regime using pulse-bursts which facilitate the formation of pores with highly circular shapes down to 1.1. We demonstrate this process for a wide range of speeds, pulse repetition rates and pulse energies, thus opening the door to a much more useful nanofabrication technique for nanophotonics. Finally, we also report the change in index of refraction that is produced at the nanoscale obtaining a positive index contrast of ∼3%. The work therefore provides a promising path towards reliable 3D nanostructuring of solid-state laser media for the flexible fabrication of large and complex structures with features sizes from the nanoscale up to the mm-scale. Moreover, due to the embedded, seamless, and monolithic nature of this technology, and since YAG crystals can sustain temperatures of up to 1900 °C and are highly chemically inert and erosion resistant, we anticipate its direct application in harsh environments.

## 1 Introduction

Current innovation in photonics, metamaterials, and nanotechnology in general is based on the spatial control of matter at the nanoscale. The development rate of novel nano-engineered structures and devices is currently larger than ever, with a wide plethora of envisaged technologies with a great potential to revolutionize our information and communication technologies. For all these proposed technologies however, the most important development phase is their experimental realisation, but current nanofabrication methods still do not meet most of the requirements imposed by either the nanostructure design or by the application in real-world harsh and even extreme environment scenarios. Indeed, real nanostructures are extremely difficult to fabricate, and need sophisticated fabrication techniques typically restricted to two-dimensional layouts. Beyond planar lithographic technologies such as those developed for the microelectronics semiconductor industry or advanced surface scanning probe ones [1, 2], great efforts have also been devoted, through different physico-chemical processing strategies, to extend the nanostructuring process to the third spatial dimension. Electron-beam-induced deposition of 3D nanostructures is now known for almost 70 years [3], focused-ion-beam chemical vapor deposition of 3D nanostructures is known since the 1990s [4, 5], and proton beam writing has also been investigated for the fabrication of 3D nanostructured objects since the 2000s [6]. Although efforts in this direction appeared to decay with time (due to the low scalability, excessive complexity, and cost of these electron- ion- and proton-irradiation approaches) recently there has been a renaissance of interest spurred by the industrial progress in these microscopies [7]. Still however, the most versatile and efficient tool to process materials in 3D was rapidly recognized to be the ultrashort pulse laser. Since 1996, when Maruo et al. first reported a method for 3D microfabrication based on multiphoton polymerization with near-infrared femtosecond (fs) laser pulses [8], 3D fs-laser nanolithography has grown to be an established technique for the multimaterial additive fabrication of functional components, from photonic bandgap polymer structures since 2002 [9], to nano-architected materials in the last decade [10, 11]. Recently, the potential of other laser-based additive 3D nanofabrication schemes such as laser-induced transfer and light-directed assembly have also been investigated for the 3D additive manufacturing of micro- and nano-structures [12]. The limitation of these 3D additive printing approaches, however, is that (1) the micro-nano-printed components are isolated and often need additional supporting trusses and frames to overcome mechanical fragility, (2) that are difficult to integrate within other heterogeneous material platforms, and (3) that the developed materials still do not have the electro-optical quality of standard industrial materials. An alternative approach to 3D laser printing has been the direct micro-nanoprocessing of the bulk optical materials of interest, such as dielectric glasses and optical crystals, to either modify their properties locally at the nanoscale, or to produce subtractive (hollow) high-contrast micro- and nanostructures. In 1997 Glezer et al. demonstrated that 3D fs-laser writing (3DLW) can produce sub-micron voxels inside optical media with diameters on the 200 nm scale, which are composed of an amorphous nucleus surrounded by a largely stressed volume [13]. This approach, termed the *microexplosion method* was investigated for some years as a route to 3D micro-nanofabrication in optical materials [14–16], but stress-induced cracking was found to be a large limiting factor for the production of quality nanostructures. In the last 20 years, large advancements have been produced in the understanding of (1) the light–matter interactions that take place when tightly focusing fs pulses to the sub-micron scale, and (2) the types of material modifications that can be achieved [17]. However, achieving an equivalent resolution and fidelity as that of 2D nanolithography in the three dimensions, has still been a hard multidisciplinary scientific challenge that is yet to be achieved.

Recently, Ródenas et al. [18] showed that the creation of hollow nanopore lattices with feature sizes at the 100 nm level and footprints on the cm scale is feasible inside a crystal in absence of crack formation and damage, this allowing to directly engineer seamless nanophotonic structures inside high quality optical crystals for the first time in 3D. The discovery of this 3D subtractive nanolithography technique has opened the door to performing sub-wavelength nano-engineering of solid-state laser crystals for the first time, anticipating the advent of a new class of solid-state media with inside-the-crystal seamlessly embedded photonic elements with designed optical responses driven by sub-wavelength light modes. Examples include dispersion-engineered photonic-crystal waveguides, sub-wavelength diffraction gratings, 100-nm thick mm-long crystalline membranes, and other structures. The technique relies on the discovery of a giant wet-chemical etching rate selectivity of up to a million (10^6^) between 3D fs-pulse laser written nanotracks and the surrounding non modified volume, forming nanopores with lengths in the mm scale and widths down to 100 nm, in hours. However, the reported nanopore fabrication laser scan speeds were too high (1–2 mm/s) to define with precision dense nanophotonic architectures with arbitrary features on the nanoscale, such as for example metalens. There is a need to fabricate these structures at low scan speeds, while maintaining the ultra-high wet-chemical etching selectivity, this implying that there is the need to understand how this nanoscale photomodification process occurs for different writing energy dose levels, i.e., for different pulse repetition rates and writing speed combinations.

In this work we show, for the first time to our knowledge, that nanopores can be formed for a wide range of laser scan speeds from µm/s up to mm/s level, and for pulse repetition rates from the 1 kHz to the 1 MHz range, this therefore opening the fabrication flexibility for a wide range of different devices with diverse footprint and complexity, and with therefore varying scan speed requirements. We also investigate how the nanopore wet-etching rate varies for different pulse energies, reporting for the first time on three differentiated wet-chemical etching rate regimes. Secondly, we investigate in detail the achievable nanopore cross-sectional aspect ratios (AR), normally in the 2 to 3 range (height along optical axis divided by transversal width), with the aim of finding conditions for which the AR can be equal to 1 which would be highly beneficial for applications such as photonic crystal structures. We show that by finely controlling the local temperature at the focal volume it is possible to drastically decrease the AR down to almost 1, obtaining quasi-circular pores of variable size, and for variable speeds and repetition rates. To accomplish this, we demonstrate the use of fs-pulse bursts as an efficient way of controlling the temperature at which photomodification occurs, depending on the pulse energy and the number of pulses within a burst. Finally, we characterize the change in index of refraction at 3DLW volumes in YAG for the first time to our knowledge, obtaining values of ∆*n*_o_ = +5.9 × 10^−2^ (±3.5 × 10^−2^), a high index change which further allows to incorporate light guiding elements with confined modes to the existing nanophotonic structures composed of hollow nano- and micro-architectures.

## 2 Experimental methods

**Figure 1:**
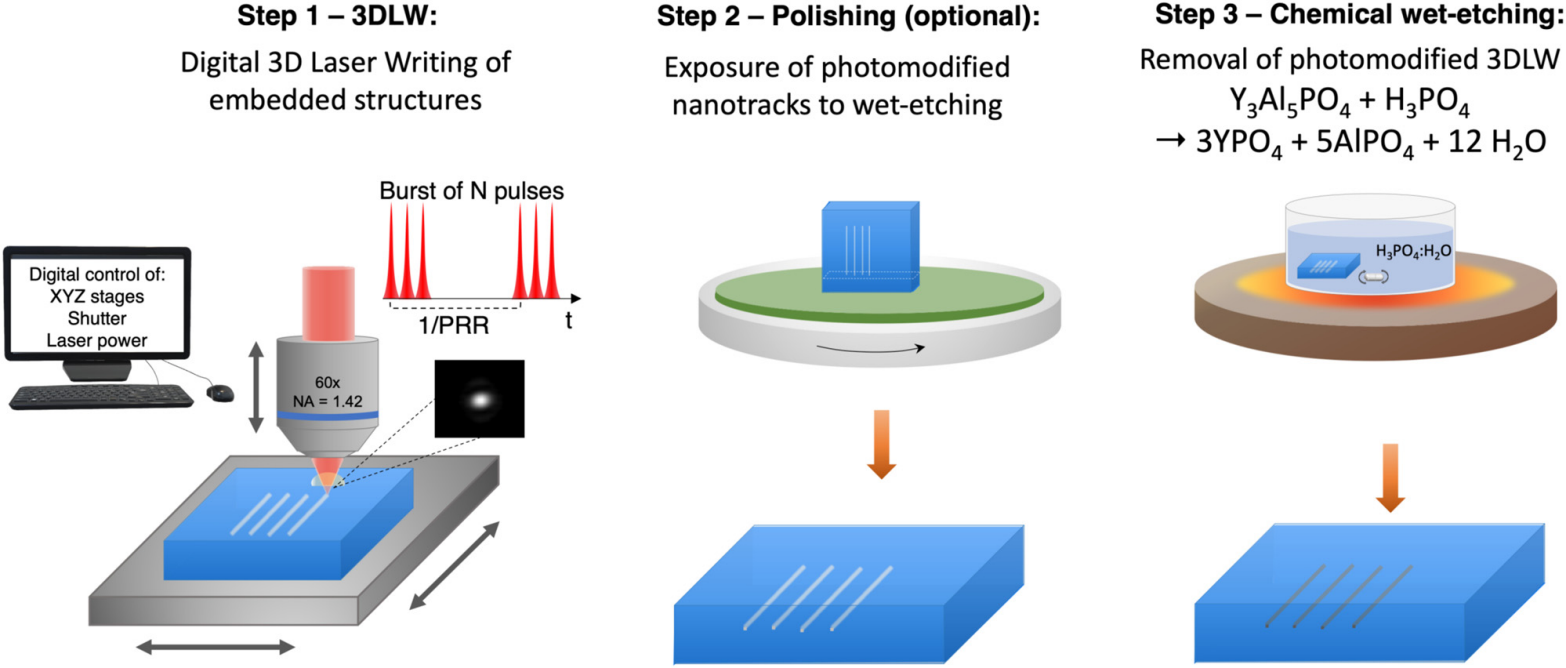
Schematic diagram of the 3D laser subtractive nanolithography process. Step 1: computer controlled 3D laser-writing (3DLW) is performed inside the sample by means of synchronously controlling several systems such as 3D nanopositioning stages, optical shutter (on/off), pulse-bursts mode, laser power, pulse repetition rate, and others. This process leaves inside the sample a 3D layout or refractive index modified nanoregions (typically positive index changes). Step 2: to wet-etch out the photomodified 3D layout a part of it must reach surface so that the chemical reaction can take place. The sample can be polished to expose some nanotracks from which etching nanopores will develop, or vertically written pores can be included in Step 1 that will serve as direct wet-etching pores without polishing. Step 3: the laser-written 3D lattices are wet-etched in phosphoric acid (H_3_PO_4_ 44 wt% solution) in deionized water with a magnetic stirrer and a digital ceramic heating plate at 350 K.

A three-step 3D digital fs-pulse laser nanolithography process is performed which does not need clean room conditions, following previous report from [18]. Figure 1 shows the schematic workflow, composed of the following three steps.

### 2.1 Three-dimensional laser writing (3DLW)

In this work, a tunable mode-locked Ti:Sapphire Tsunami (Spectra-Physics) fs laser was used, tuned to 804 nm wavelength, 120 fs pulses, with a maximum pulse energy of ∼20 nJ and with a repetition rate of 80 MHz. To decrease the pulse repetition rate down to 1 MHz or lower values, as well as to perform a burst-mode writing with controllable intra-burst pulse numbers, an ultrafast pulse picker UP2 (Eksma Optics) was used, with a digital synchronization and delay generator pMaster 4.2. Linearly polarized pulses from the laser were tuned in energy by means of a half-waveplate and a polarizer, and circular polarization was produced and used for 3D writing placing a quarter-waveplate before the focusing objective. Tight focusing was obtained using a PlanApo N 60X 1.42NA Olympus objective. All pulse energies here reported are calculated considering the transmission of the whole 3DLW optical system including the objective. XYZ nano-positioning stages (Aerotech Ltd.) were also used to control the optical writing in 3D inside undoped YAG crystals, with no defined crystallographic orientation in this work since YAG has an isotropic refractive index distribution.

### 2.2 Exposure of photomodified tracks

An intermediate step between 3DLW and wet-chemical etching is required if the written nanotracks are all inside the crystal far from its superficial faces. To initiate the wet-etching reaction either polishing of the sample is performed, to expose laser written tracks to the sample surface, or vertically written tracks which start from surface to the written lattice, are included that allow to wet-etch the 3DLW structure directly without lateral polishing. Optical polishing is a mechanical process that can be done easily and only requires that some tracks are written closer to the sample facets, so that polishing is a fast and simple process leading to optical quality surface. Polishing the sample until the written tracks are exposed to air has the advantage of facilitating the study of the cross-section morphologies of the written structures by SEM. In the case of writing vertical pores to serve as inlets for the acid instead, these extra pores serve to selectively etch larger volume of written structures within the crystal and can also be utilized to further engineer the device architectures with local 3D control inside the sample. See further experimental results and data on this approach in the Supplementary Information from [18].

### 2.3 Wet-chemical etching

The 3DLW structures inside YAG crystals are wet chemical etched in phosphoric acid (H_3_PO_4_ 44 wt% solution) in deionized water with a magnetic stirrer and a digital ceramic heating plate at 350 K. The chemical reaction that could explain the observed wet etching of nanopores is: Y_3_Al_5_O_12_ + 8H_3_PO_4_ → 3YPO_4_ + 5AlPO_4_ + 12H_2_O [18].

## 3 Results and discussion

### 3.1 Standard 3DLW nanolithography at 1 MHz pulse repetition rate

The laser experimental setup here used, composed of a 80 MHz Ti:Sapphire mode-locked fs pulse laser with an external ultrafast pulse picker, allowed us to first investigate the fabrication of nanopores at similar conditions (in terms of writing speed and pulse repetition rate) to those seminally published in [18], but with a shorter fs-pulse duration (120 fs against 350 fs) and a shorter pulse wavelength (804 nm against 1030 nm). The effect of shorter fs-pulses in the non-linear interaction is yet unknown, but similar results may be expected since the same focusing objective, polarization state, scan speed, repetition rate and pulse energies, are all used. This first study therefore sets the basis for the rest of the present work, in which pulse-bursts are introduced that change the local temperature during photomodification.

#### 3.1.1 Nanopore cross-sections

**Figure 2:**
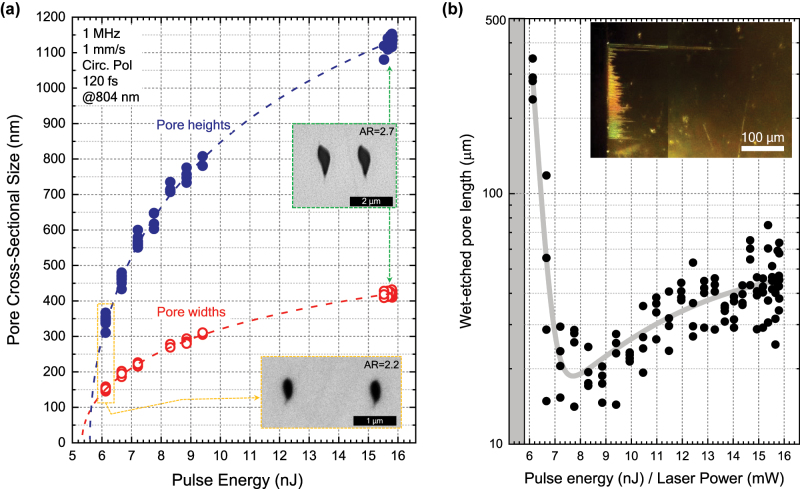
Dependence of the hollow nanopore widths and heights with laser pulse energy for the laser writing parameters indicated in the figure inset (a). The aspect ratio (AR) varies from 2.2 at the minimal pulse energy measured to produce nanopores, to 2.7 for excessive pulse energy. Logarithmic fitting of data indicates that the rate at which pore heights diminishes is faster than that of the pore widths, this explaining the decrease in AR, which could reach AR = 1 if precise threshold pulse energy could be reliably applied (beyond the pulse-to-pulse stability of the laser used in this study). The wet-etching length of nanopores for 15 h of etch bath is shown in (b), as a function of pulse energy. Two different regimes are observed, maximal etch rate is observed at minimum pulse dose, lowest etch rate at an intermediate energy, and a plateau value for high pulse energies is also observed. Inset shows a dark field image of light scattering from etched nanopores.

The cross-sectional dimension of nanopores was first identified for this non-thermal regime for 1 MHz pulse trains. Scanning electron microscopy (SEM) was performed to measure the widths and heights of nanopores as a function of pulse energy, as shown in Figure 2(a). The inset in Figure 2(a) shows the experimental laser writing conditions, and two SEM images of pores formed at high and low pulse energy, with different cross-sectional size and with aspect ratios of 2.7 and 2.2, respectively.

The evolution of both pore width and height with pulse energy can be fitted to a linear relationship between the square of these and the logarithm of the pulse energy, supposing in first approximation Gaussian point spread functions at the focus [19, 20]. That is, $D^{2}=2\omega_{0}^{2}\ln\;(E_{\text{pulse}}/E_{\text{th}})$ , where *D* is the measured diameter of the photomodified volume across the measured direction (either vertical for the height or horizontal for the width), *ω*_0_ is the effective focal radius of the 3DLW setup in this non-thermal conditions, *E*_pulse_ is the pulse energy used, and *E*_th_ is the pulse energy photomodification threshold for which *D* = 0. As shown in Figure 2(a), the trend of the pore height decreases faster towards lower energy than that of the pore width and presents a pulse energy threshold *E*_th_ of 5.6 nJ, which is slightly higher than that obtained for the width, which is of 5.3 nJ. Both fittings had corresponding *R* ^2^ = 0.997. These different trends towards the threshold indicate that indeed the aspect ratio is not constant towards the threshold, as it was also found in [18]. From the data in Figure 2(a) we observe that the aspect ratio of pores remains constant at around 2.7 from high pulse energy down to around 7 nJ pulse energy, and only below this value it diminishes noticeably (see also Figure 4). From the fitting, the effective radii *ω*_eff_ of the photomodification volume cross-sections are obtained, being of *ω*_eff_ = 285 nm for the radius across the horizontal *XY* focal plane, matching well with the theoretical beam radius for a 1.42NA lens and 804 nm wavelength (*ω*_o_ = 0.61*λ*/NA = 345 nm), and of 786 nm along the optical axis *Z* (*ω* ^*z*^_eff_), matching well with the theoretical confocal parameter ( $\omega_{z}=\pi \omega_{o}^{2}n_{\mathrm{Y AG}}/\lambda =849\;\;nm$ ). The approximate theoretical aspect ratio is therefore of 2.5, close to the experimental value of 2.7 for medium pulse energy pores.

#### 3.1.2 Nanopore wet-chemical etching rates

Figure 2(b) shows the nanopores lengths after wet-chemical etching for 15 h. The inset shows a dark field microscope image where the scattering from hollow pores is observed. Two clear trends are observed: a fast exponential increase in wet-etch rate towards lowest pulse energy pores (also lowest pore cross-section and AR) from around 8 nJ, where a minimum in etch-rate is observed, and a slow increase in etch rate towards saturation plateau at higher pulse energies from this minimum value. The grey line in Figure 2(b) is included as a guide for the eye and was obtained by fitting the data to two exponential functions. Coincidentally (for this fabrication conditions at 1 MHz and 1 mm/s scan speed) at around 7–8 nJ both the AR of pores and its wet etching rate, have a marked change in trend. This pointing towards a differentiated physical process undergoing only at close-to-threshold pulse energy, which is both involving a confinement of the photomodified volume along the optical *z*-axis as well as boosting the wet-chemical etching rate.

The nature of the photomodification in fs-3DLW YAG crystals, which leads to enhanced wet-etching selectivity, was first experimentally identified as a localized lattice distortion in absence of bond breaking (i.e., localized strain) which increases chemical reactivity [21]. The presence of nanocracks was also pointed out as a vector favoring acid penetration [21]. At the light of this the complex behavior observed in Figure 2(b) can be hypothetically explained as follows: at low pulse energy, pore sizes are the smallest, which could entail a higher chemical reactivity from a pure confinement effect linked to a high stress state within the localized volume of written nanotracks. As the pulse energy is increased, the decrease in etch rate could be explained both by a larger pore cross-section where chemical reactivity enhancement is weaker and by a lower stress state in the case that nanocracks start to occur, releasing the stress which drives reactivity. Beyond the minimum etch rate point at around 8 nJ, nanoconfinement effects could no longer be relevant, but a higher density of nanocracks and stress material could still provide a reactive path, so that the reaction slowly increases towards a plateau. This behavior is also similar to that previously studied in 2010 by Rajesh and Bellouard [22], for large micrometer damage tracks in fused silica glass. Further investigation of these dynamics is outside of the scope of the present manuscript, but it is to be examined in a forthcoming work.

### 3.2 Nanopore aspect ratios in the non-thermal accumulation regime (PRR ≤ 1 MHz)

Up to now [18], it is known that almost circular nanopores can only be achieved for small nanopores of 100 nm fabricated at the threshold condition. However, at the threshold for photomodification the pore cross-sectional size and shape has a strong dependance with pulse energy, and it was observed that only a slight +0.2 nJ increment in pulse energy produces a 2-fold increase of the AR [18]. This poses two main limitations for fabricating nanophotonic systems with this technique: First, a very fine control of pulse energy is needed, and a high pulse-to-pulse stability of the laser system is as well also needed. For the strong dependance of AR with pulse energy that was observed in [18], the pulse energy deviation needs to be within ±0.01 nJ along the full fabrication time; this corresponding to ∼0.15% stability at threshold pulse energy (∼6 nJ). In the present work, the pulse energy precision of the mode-locked Ti:Sapphire based 3DLW system was for example of ±0.2 nJ, not enough to work at the tight threshold conditions. Secondly, the threshold condition also implies a fixed pore size of around 100–120 nm, this greatly limiting the potential of the lithographic technology, as for example for the fabrication of photonic crystal fiber-type waveguides circular pores with variable diameter, and larger than 1 µm diameter, are usually needed. As a result, the fabrication of circular pores with larger diameter than 100 nm has hitherto been impossible.

In this section we investigate the formation of nanopores and their ARs, across a very wide range of 3DLW parameters, with pulse repetition rates from 1 kHz up to 1 MHz. This range is calculated to produce no thermal accumulation between pulses, by calculating the heating between subsequent pulses for up to 1 MHz pulse trains, for the tight-focusing experimental conditions used in this work (see Section 3.3 for further details).

#### 3.2.1 Nanopore ARs as a function of pulse energy

**Figure 3:**
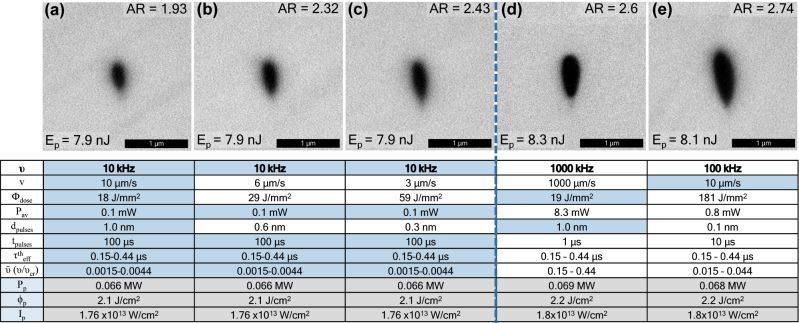
Nanopores fabricated for fixed pulse energy of ∼8.0 nJ (±0.2 nJ) and three different pulse repetition rates: 10 kHz (a–c), 1000 kHz (d) and 100 kHz (e), with various other varying parameters. The table shows corresponding parameters: scan speeds, pulse dose (J/mm^2^), average power, distance between pulses along scan, pulse to pulse arrival time, effective cooling time, normalized repetition rate, pulse peak power, pulse mean fluence, and intensity. The nanopore’s ARs are indicated in the figure insets, increasing from left to right, from 1.9 to 2.7 for the fixed pulse energy.

Figure 3 shows SEM images of five different nanopores all fabricated with the same pulse energy of 8.0 nJ (±0.2 nJ) but different pulse repetition rates of 10 kHz (a–c), 100 kHz (d), and 1 MHz (e), and speeds from µm/s scale up to mm/s. We demonstrate that nanopores can be created almost independently of the pulse rate both temporally and spatially, as far as there is no thermal accumulation between pulses. The table in Figure (3) shows eleven fs-pulse 3DLW parameters which can be determined for a systematic comparison between these fabrication regimes: the pulse repetition rate (*ϑ*), the linear scan speed (*v*), the energy dose (Φ_dose_), which is defined following [22] as the total pulse energy deposited per surface element equal to the effective nonlinear spot area (*πω*_eff_^2^) along a line scan ( $\Phi_{\text{dose}}=\left(E_{p}/\pi \omega_{\text{eff}}^{2}\right)\left(2\omega_{\text{eff}}\vartheta /v\right)=2E_{p}\vartheta /\pi v$ ), the total average power (*P*_av_), the distance between deposited pulses along the scan (*d*_pulses_ = *v*/*ϑ*), the time between incoming pulses (*t*_pulses_), the effective cooling time of the focal volume after each pulse ( $\tau_{\text{eff}}^{T\;h}={\left(2\omega_{\text{eff}}\right)}^{2}/D_{\text{th}}$ ) [23], where *D*_th_ is the thermal diffusivity of YAG (*D*_th_ = *κ*/*ρc*_*p*_), where *κ* is the thermal conductivity, *ρ* is the density, and *c*_*p*_ is the specific heat capacity; for this study, we used *ρ* = 4576 kg/m^3^, *c*_*p*_ = 600 J/kg K, and *κ* = 3 W/mK, supposing a temperature at the center of the focal volume of 1500 °C [24], well below the melting temperature of YAG (∼1940 °C). Additional parameters in the table include the normalized repetition rate ( $\bar{\vartheta }$ ), corresponding to the laser repetition rate normalized to the critical one at which thermal accumulation between pulses is approximately expected to occur ( $\vartheta_{cr}=1/\tau_{\mathrm{eff}}^{th}$ ), the pulse peak power (*P*_*p*_), the peak fluence supposing a Gaussian distribution at focus (*ϕ*_*p*_), and the peak pulse intensity (*I*_*p*_). These three last parameters, peak power, peak fluence, and peak intensity, are equal for all pores in Figure 3, since pulse energy, duration, polarization (circular) and focusing optics are always the same. Therefore, only parameters related to temporal and spatial characteristics of the pulse irradiation are therefore differing in the 5 cases.

**Figure 4:**
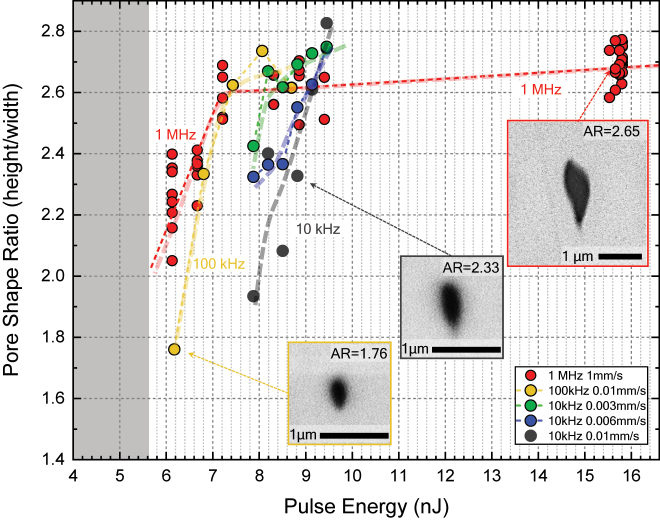
Analysis of pore shape aspect ratio against pulse energy, for repetition rates of 1 MHz, 100 kHz, and 10 kHz (for three different scan speeds). Insets show representation SEM pictures of pores and middle and extremes of the analyzed range.

For a fixed repetition rate (10 kHz), Figure 3(a)–(c) show nanopores with increasing aspect ratio, from 1.9 to 2.4. In these three cases only the scan speed, pulse dose and distance between pulses varies (varying parameters are shown in white in the table), these three parameters being directly correlated. A direct conclusion could be that for a fixed repetition rate and pulse energy, the faster the scan (or equally the lower the dose) the smaller the AR, and the smaller the pore size. This important observation also holds true if we fix the speed (10 µm/s), but increase the repetition rate (i.e., the dose) by an order of magnitude, from 10 kHz (Figure 3(a)) to 100 kHz (Figure 3(e)). In this case the aspect ratio increases from 1.9 to 2.7. However, dose alone does not define the nanopore AR, as pores fabricated with same dose of ∼18.5 J/mm^2^, in Figure 3(a) and (d), and hence the same distance between pulses (1 nm), have different ARs of 1.9 and 2.6, respectively. The reason for this could be that the pulse frequency varies by 2 orders of magnitude (10 vs. 1000 kHz), and therefore the arrival time between pulses varies from 100 µs down to 1 µs. We therefore conclude that from this small data set in Figure 3, a full picture of the nanopore AR dependence of 3DLW parameters cannot be obtained.

In Figure 4 we plot the pore aspect ratios as a function of pulse energy, for repetition rates from 10 kHz up to 1 MHz. Data for 1 MHz is the same in Figure 4 as that in Figure 2. The figure shows how pulse energy undoubtedly drives the ARs as we had previously discussed from Figure 2, but it also shows that decreasing the repetition rate (i.e., increasing the time between arriving pulses), moves the threshold upwards towards higher pulse energies. To better understand this, in the next sections we analyse the data separately, in terms of scan speed and pulse energy first, and energy dose and pulse energy afterwards.

#### 3.2.2 Effect of scan speed on nanopore ARs

**Figure 5:**
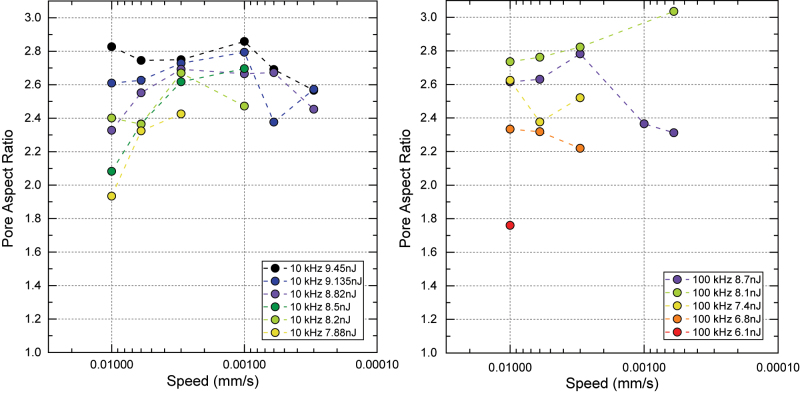
Analysi s of pore aspect ratio for different scan speeds spanning two orders of magnitude, and for different pulse energies. Left and right figures correspond to 10 and 100 kHz repetition rates, respectively. Color scale is related to pulse energy, and it is the same for both figures.

To analyze the effect of scan speed on pore aspect ratio, in Figure 5 we separately plot pore ARs against speed for different pulse energies and pulse repetition rates. General trends for scan speed are not uniform across different pulse energies and repetition rates, and with the available data it is not clear to infer a general trend.

#### 3.2.3 Nanopore ARs as a function of energy dose

**Figure 6:**
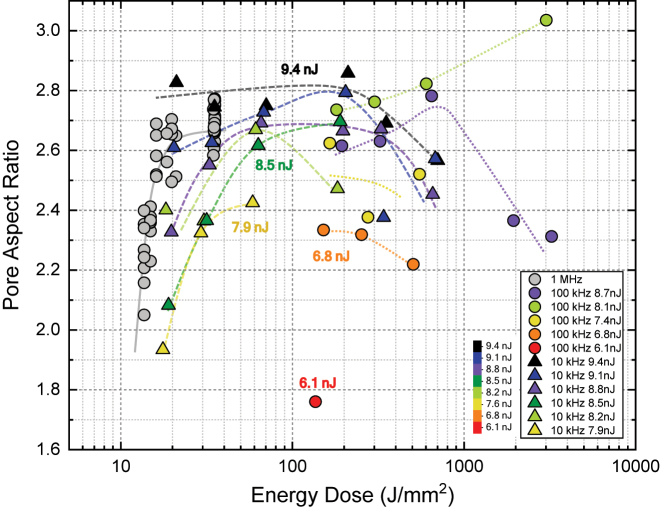
Distribution of pore aspect ratio as a function of energy dose. Color scale is indicated corresponding to the pulse energy. Point data shape corresponds to pulse repetition rate.

Figure 6 shows the pore aspect ratio against energy dose. The presented data spans two orders of magnitude of irradiation energy doses from 13 J/mm^2^ up to 3000 J/mm^2^. Three clear trends are observed in Figure 6: (1) the distribution of pore ARs appears to be centered on a value of ∼100 J/mm^2^ for which AR presents a maximum and fabrication at higher or lower doses yield lower AR. This behavior seems independent of repetition rate, pulse energy and scan speeds. (2) Pulse energy determines the maximum AR achievable independently of pulse repetition rate and scan speed. Data for different repetition rates but similar pulse energy follow similar trends. (3) The minimum achievable AR is of around 1.8, at threshold pulse energy of 6 nJ. In terms of application in nanofabrication, it is therefore discovered that if small pores are needed with minimal AR, energy doses should be kept low at around 13–30 J/mm^2^. If, however large pores with minimized AR are needed, then energy doses should be chosen above 200 J/mm^2^ towards 500 J/mm^2^. Lastly, a fabrication with constant pore AR but with a programmable pore size seems also feasible, by using a route of pulse energy and scan speed giving constant AR but larger pore size towards larger energy dose. An aspect ratio of around 2.5 would yield the larger available range of pore sizes (energy doses), under the experimental conditions given in this work.

### 3.3 Nanosecond bursts effect on nanopore aspect ratio

#### 3.3.1 Nanosecond bursts for 3D localized thermal control

In the previous sections we have shown that nanopore ARs cannot be lower than ∼1.8 if working on the non-thermal regime, and these lower values can only be achieved by working as close to photomodification threshold as possible, which depends on the pulse energy precision and accuracy of the used 3DLW fs-pulse system, as discussed in Section 3.2. In the following, we show results on the effectiveness of nanoprocessing at high (500 °C) to extreme temperatures (∼1800 °C), always below the melting temperature of the crystal to avoid melting and bond-breaking around the laser modified volumes. To produce those temperatures with accuracy, and for a controlled duration on the µs scale, we use pulse-bursts at the fundamental rate of 80 MHz within a repetition rate of 1 kHz which ensures a complete cool down to room temperature between bursts. In this scenario we can tune the heating dynamics by changing both pulse energy and pulse number. To model this heating process of the photomodified focal volume, we calculate the temperature distribution in time using the thermal diffusion equation [25]:

(1) $$\begin{array}{ll} & \Delta T\left(x,y,z,t\right)\\ & \;=\frac{E_{p}A}{\pi \left[\ln\left(2\right)/2\right]\rho c_{p}\left(w_{0}^{2}+8D_{\text{th}}t\right){\left(l^{2}+8D_{th}t\right)}^{1/2}}\\ & \;\;\times \exp\left[-\frac{2{\left(x-x_{0}\right)}^{2}+2{\left(y-y_{0}\right)}^{2}}{w_{0}^{2}+8D_{\text{th}}t}\right]\\ & \;\;\times \exp\left[-\frac{2{\left(z-z_{0}\right)}^{2}}{l^{2}+8D_{\text{th}}t}\right] \end{array}$$

where *E*_p_ is the laser pulse power, *A* is the absorptivity, *ρ* is the material density, *c*_*p*_ is the heat capacity, *D*_th_ is the thermal diffusivity, *ω*_0_ and *l* is the focus spot radius in the *XY* plane, and along the *Z* optical axis, respectively, and *t* is the time elapsed after the instantaneous pulse irradiation. The value for the absorptivity *A* was set empirically to a 2%, by experimentally noting the pulse energy threshold at which melting is seen to occur during 3DLW writing and setting it to match this experimental threshold. For this model, a Gaussian heat source was assumed. For a pulse-burst mode irradiation the temperature evolution of the sample was calculated as:

(2) $$T\left(x,y,z,t\right)=T_{R}+\overset{N-1}{\underset{i=0}{\sum }}\left\{\begin{array}{cc} \Delta T\left(t-it_{p},x,y,z\right),\; & t\geq it_{p}\\ 0,\; & t<it_{p} \end{array}\right.$$

where *N* is the number of pulses in the burst window, *t*_*p*_ is the temporal pulse spacing (12.5 ns), and *T*_*R*_ is the room temperature. For the thermal conductivity *k,* a function with temperature was used of the form:

(3) $$k\left(T\right)=\alpha +\beta \exp\left[-\left(T-T_{R}\right)/\gamma \right]$$

where *α* = 2.93, *β* = 5.83, *γ* = 356, are obtained from fitting the data from [24]. The rest of parameters are computed using the values previously discussed in Section 3.2.1, noting that we consider density and specific heat capacity as constant.

**Figure 7:**
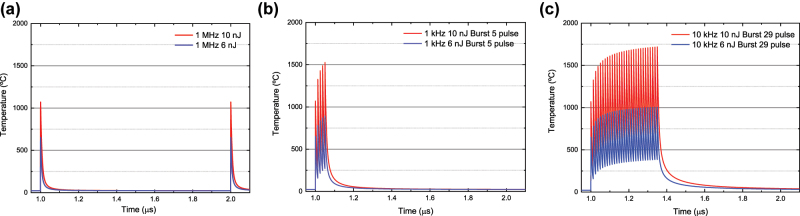
Calculated temperature evolution at the center of the focal volume for two different pulse energies: threshold pulse energy of 6 nJ, and a high pulse energy of 10 nJ. Temperature evolution is shown for 1 MHz pulse train (a), 1 kHz and 5 pulse bursts (b), and 1 kHz 29 pulse bursts, three 3DLW configurations in which no melting can occur, as also confirmed experimentally.

Figure 7 (a)–(c) shows the temperature temporal distributions calculated for the centre of the focal volume, for three experimental conditions studied in this work. The calculations are performed for threshold pulse energy (6 nJ) and for a high pulse energy of 10 nJ. Figure 7(a) first shows the local heating for a single-pulse 1 MHz pulse train, corresponding to the experimental conditions studied in the previous sections in this work. As it can be seen, at 1 MHz the temperature at the center of the focal volume decays very fast to room temperature (23 °C) within fractions of a µs. Peak temperatures for 1 MHz are of 650 °C and 1070 °C for threshold (6 nJ) and high (10 nJ) pulse energies. This confirms that fabrication with repetition rates of around 1 MHz or lower do not produce thermal accumulation between pulses, and thermal dissipation occurs extremely fast within ∼100 ns. The main cause for this ultrafast heat dissipation is the tight focusing conditions achieved with 1.42NA focusing lens and producing a sub-micron focal volume which dissipates heat very fast. To achieve a fine temperature control, a pulse-burst with 5 pulses (12.5 ns pulse to pulse time) is shown in Figure 7(b) to induce local heating of up to 900 °C for 6 nJ pulses, and 1530 °C for 10 nJ pulses. Thermal cool down to room temperature also takes around ∼200 ns. Lastly, in the case of 29 pulse bursts, shown in Figure 7(c), thermal recovery to room temperature takes almost 1 µs, and peak temperatures are of 1000 °C for 6 nJ threshold pulse energy, and 1700 °C for 10 nJ. In the next sections we report how nanofabrication under these localized heating conditions modifies the pore aspect ratios and its dependence of irradiation energy dose.

#### 3.3.2 Nanopore aspect ratio reduction by nanosecond burst control

**Figure 8:**
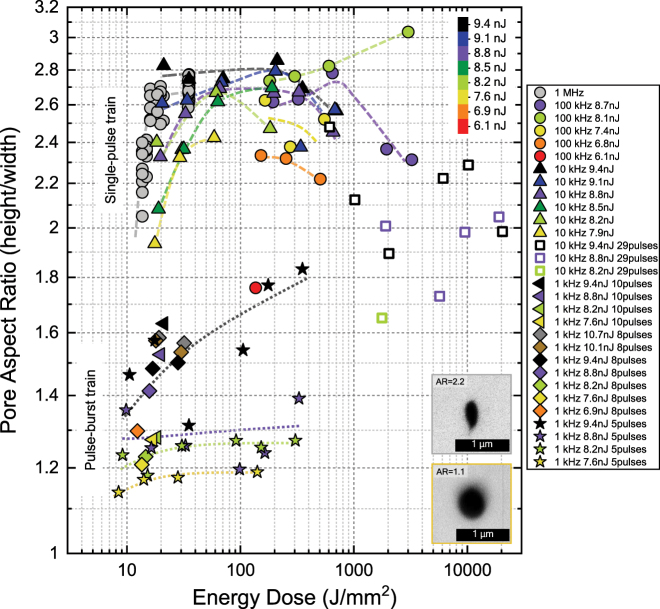
Nanopore aspect ratio as a function of energy dose, for both the non-thermal 3DLW regime (single-pulse train) and the thermally controlled one (pulse-burst train), as shown in Figure 7. Inset shows examples of pores fabricated at each regime, showing how temperature control allows expanding the photomodification distribution in the orthogonal plane to the 3DLW *z*-axis.

Figure 8 shows the aspect ratio of pores fabricated at both single-pulse and pulse-burst trains, as a function of energy dose. Dose is calculated as previously explained and considering the number of pulses per burst. Inset shows an example of two nanopores fabricated at minimum pulse energy with a single-pulse 1 MHz repetition rate at a dose of ∼13 J/mm^2^, and a nanopore fabricated with 5-pulse bursts and a threshold dose of ∼9 J/mm^2^. The data color scale follows the same criteria as in Figure 6. The range of doses which produce wet-chemical etched nanopores appears to match that of single-pulse trains, with the only apparent difference that the minimal dose threshold appears to lower down, this pointing to a more efficient non-linear energy transfer from fs-pulses to the photomodified lattice state which induces the wet-chemical etching, as discussed previously in Section 3.1.2 corresponding to highly stressed volume with minimal density of nanocracks.

A clear improvement of the 3D nanolithographic technique is observed on three important aspects simultaneously. First, nanopore AR values decrease from 1.8–2.8 values down to 1.1–1.6. i.e., it is reported that it is possible to fabricate pores with variable cross-section size but ARs close to 1.1. The data also suggests that fully circular pores may be fabricated if lower pulse energies than 7 nJ are tested with a 3DLW system with higher pulse-to-pulse stability than the one here reported. Second, for small ns-bursts of 5 pulses or less and pulse-energies of 8 nJ and below, the AR appears to become independent from scan speed, and therefore from dose. This being a very important feature for nanofabrication that is not observed in the case of single-pulse trains. For example, in the whole dose range from 8 J/mm^2^ to 300 J/mm^2^, the AR of nanopores written at 7.6 nJ does not vary from 1.14 to 1.19, this being another important improvement in terms of robustness and fidelity of the 3D lithographic technique. Third, burst pulse number appears as a powerful parameter which can be easily tuned on-the-fly during fabrication and allows changing pore shape and size, in cases where scan speed or pulse energy or the dose needs to be fixed. Such as for example when scan speeds need to be very low to maximize the 3D design fidelity on the nanoscale when fabricating complex architectures with details on the nanoscale but footprints on the microscale or larger. At 5-pulses per burst, a regime where AR seems to be very stable is found, and the dose threshold also appears to slightly decrease from the case of non-thermal regime with single-pulse trains.

#### 3.3.3 Burst pulse-number effect on pore size and shape

**Figure 9:**
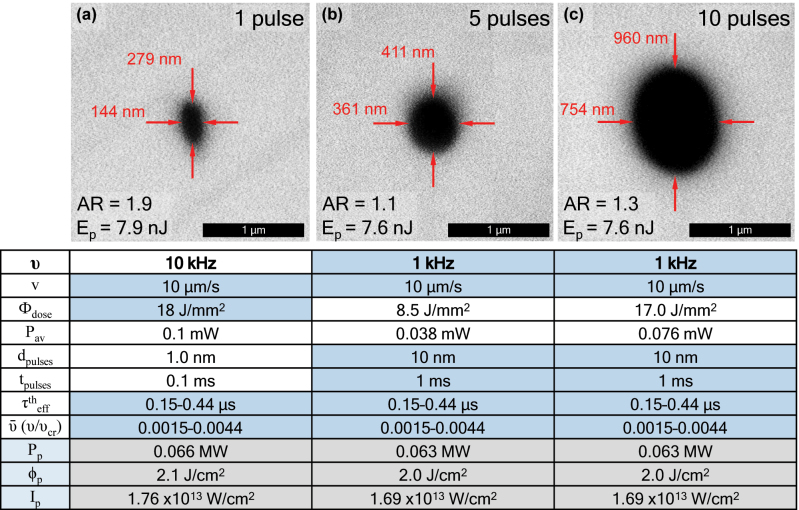
Pores fabricated with fixed pulse energy of ∼7.7 nJ and equivalent threshold energy doses, and different burst pulse numbers of 1, 5, and 10. The aspect ratio is shown to dramatically improve from almost ∼2 down to ∼1 for few-pulse bursts.

The effect of burst pulse number at close-to-threshold conditions (7.7 nJ ± 0.2 nJ) is also depicted in Figure 9. The pore cross-section is shown for 1 pulse bursts, 5 pulse bursts, and 10 pulse bursts, in Figure 9(a)–(c), all written at the same speed of 10 µm/s. The aspect ratio (AR) is shown to vary abruptly from ∼1.9 down to ∼1.1 when adding 5 pulses. The further addition of pulses up to 10 induces, however, a slight increase of AR to 1.3. Nanopores with almost circular shape and cross-sectional diameter of around 400 nm were previously impossible to fabricate with single-pulse trains, this opening a door to fast 3D lithography with symmetric structures. It is convenient to recall here that achieving perfect AR = 1 is technically possible by performing multiscan with the required lateral offset to match horizontal width with vertical height [18].

### 3.4 Characterization of refractive index change at 3DLW nanotracks

In the previous sections we have characterized the pore wet-etching dynamics and aspect ratio shapes. A fundamental aspect of the 3DLW technique for optics and nanophotonics fabrication is that fs-pulse 3DLW can induce positive index changes (+Δ*n*_o_) which can be used to fabricate photonic devices with 3D architectures impossible by other photonic fabrication techniques. The inscription of embedded positive index-change 3D architectures in optical crystals has been already studied with microscale (∼10 µm) structures, with the first reports showing that it is possible to produce step-index waveguides with refractive index changes of around 10^−3^, with confined modes in various crystals such as YAG [26], lithium niobate [27], ZnSe [28], and YCOB [29], and recently with demonstrated application in quantum photonics [30, 31]. The type of index change produced on the nanoscale if 3DLW nanotracks, has however never been reported. To measure the Δ*n*_o_ at photomodified regions we use the wavefront phase imaging technique (WFPI), a quantitative phase imaging (QPI) technique [32] that has been applied before in microscopy and another fields as metrology or ophthalmology [33, 34], and it is a non-interferometric approach based on mechanical defocus [35]. The experimental setup consisted of an inverted Olympus CKX53 microscope (60X Plan C Achromat Dry Objective. NA 0.8) with a custom motorized focusing system that can move vertically with an accuracy of 500 nm, and a collimated LED as the illumination source (*λ* = 470 nm). Defocus distance *d* (distance between the two planes that images are recorded) was optimized experimentally, seeking a compromise between lateral resolution (worse as the defocus distance increases) and SNR (better as the defocus distance increases). The optimum *d* was chosen as 24 µm. Images were recorded in ASI1600-ZWO camera, equipped with a high-resolution CMOS sensor with a pixel size of 3.8 µm. The final lateral resolution of the system, limited by the defocus distance, was around 1.7 µm and the axial *Z* sensitivity, obtained experimentally by BIAS statistical analysis, was 5 nm.

**Figure 10:**
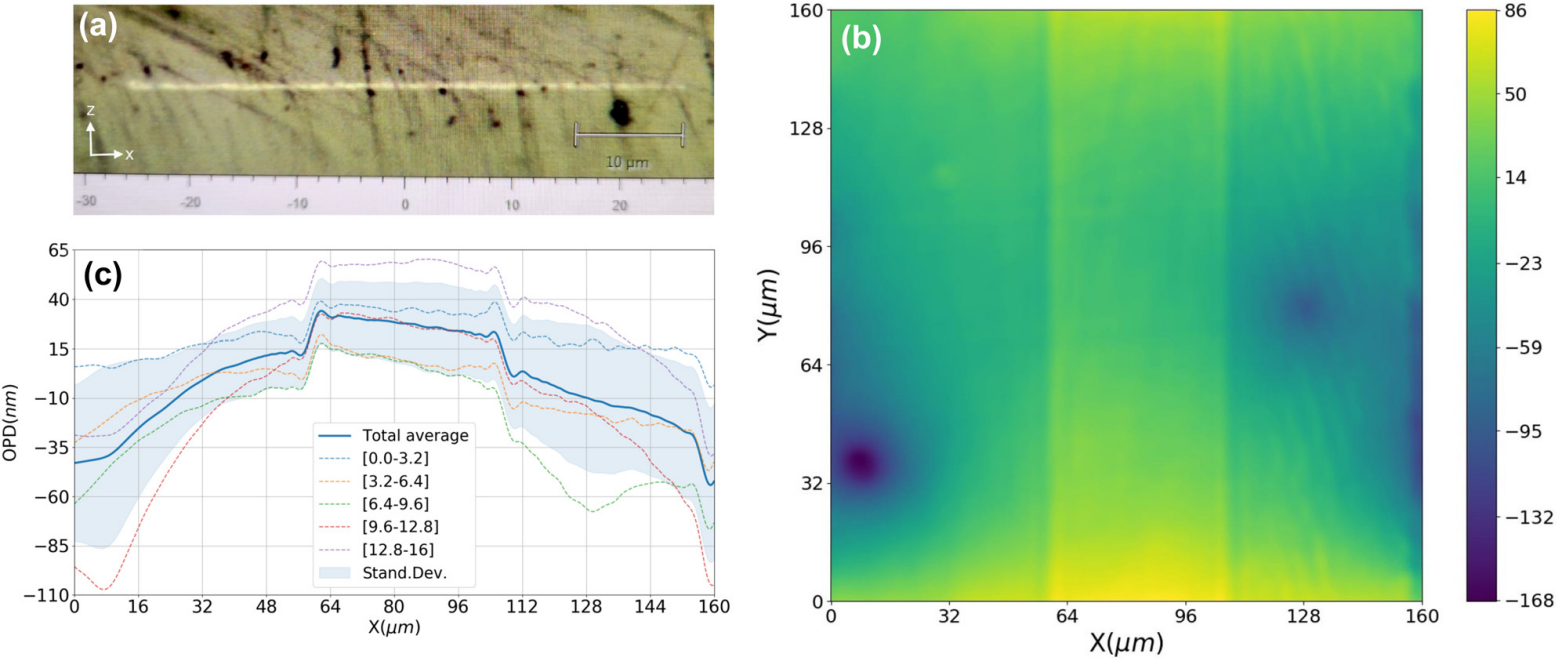
Measurement of optical path difference (OPD) at 470 nm wavelength by WFPI technique across a 300 nm thick (along *z*) photomodified nanoplane which is 50 µm wide (along *x*) and 1 mm long (along *y*). A lateral reflection white light microscope image of the nanoplane facet after mechanical polishing is shown in (a), which shows the reflective nature of the photomodified region. The OPD image is shown in (b), and the step changes in OPD profiles are shown in (c), as discussed in the text.

A continuous plane of width 50 µm and 1 mm length, was written with a 3-pulse burst 1 MHz pulse train, at 1 mm/s, 6 nJ pulse energy, and a separation between tracks of 25 nm in zig-zag mode, so that the plane is homogeneously composed of nanotracks. The height Δ*z* of the plane was measured to be 300 nm (±10 nm). Figure 10(a) shows an optical reflection microscope image, which clearly shows the reflective nature of this nanoplane, indicating that at least in the visible range the index change is positive. Figure 10(b) shows the OPD map obtained by WFPI in an intermediate zone of the plane, where a clear positive step is obtained within the inscribed plane area. To analyze the optical path length change at the plane, the average profile along the *x*-axis is calculated, shown in Figure 10(c). In addition, Figure 10(c) shows the standard deviation and averages for sub-intervals on the *y*-axis. As can be seen, the average profile captures the lithographed area in the form of a plateau. Furthermore, from the partial averages and the standard deviation, the OPD profile changes significantly depending on the region of the *y*-axis, which is chosen, this being due to contamination of the optical system and/or irregularities at the optically polished external faces of the crystal. On the other hand, there is a standard deviation ’offset’ that affects all the *x*-axis profiles equally, due to the instrumental error, equal to ∼16 nm, which is also accounted for. Besides this, the standard deviation is seen to be lower at the central region where the plane is positioned. To calculate the OPD due only to 3DLW, we do the integral average for all *y*-axis of the OPD difference from a point within the plane (*x* = 67 µm) and a near point outside the modified region (*x* = 54 µm), approximating this integral by a Riemann sum due to minimal size of the sampling within the CCD detector. An OPD of 18 nm (±10 nm) is obtained by considering the maximum error, where the error is taken with simple error propagation of the instrumental error (5 nm). From this OPD and considering the Δ*z* of the plane, we obtain a value of ∆*n*_o_ = +5.9 × 10^−2^ (±3.5 × 10^−2^), a high positive index-change never reported before for nanostructures, to our knowledge, in 3DLW crystals. Since the index of YAG is of 1.846 at 470 nm, the index contrast (Δ*n*/*n*_o_) of these photomodified volumes is of 3.2%, significantly higher than previously reported inscribed contrast in a crystal by fs-pulses to our knowledge (0.67% [29]), and in a finite region which is 300 nm in extension (along optical path), while previous photomodified regions were of around 7 µm size [29].

## 4 Conclusions

In this work we have shown for the first time to our knowledge that 3D fs-pulse laser nanolithography with almost circular nanopores can be performed in YAG optical garnets if controlled pulse bursts on the nanosecond scale are used, a feature hitherto impossible by the simple resolution limit of focusing optics dictated by the Abbe principle. This new finding now allows to nanostructure solid-state YAG laser crystals with pores from 100 nm width up to 1 µm, and almost circular cross-section, for length on the mm to cm scale. Furthermore more, compared to previous reports where laser scan fabrication speed was of 1–2 mm/s, we now report an extensive study proving that speeds down to µm/s can also be used, and repetition rates in the whole range from a few pulse up to the MHz ranges are also feasible, this greatly opening the range of optical systems that can be used, and more importantly, the range of devices which can be fabricated with complex features on the nm scale and large footprints on the mm to cm scale. In addition, we also report how the nanopore wet-etching rate varies for different pulse energies, reporting for the first time on three differentiated wet-chemical etching rate regimes. Finally, we have also characterized experimentally an important feature of 3DLW with great application in 3D photonics, which is the change in index of refraction of the nanoscale photomodified regions, obtaining a value of ∆*n*_o_ = +5.9 × 10^−2^ (±3.5 × 10^−2^), a positive index change which further allows to incorporate light guiding elements with confined modes to the existing nanophotonic structures composed of hollow nano and micro-architectures. Concerning other types of crystals, previous work [18] has shown that nanopores can also be performed in sapphire crystal with the same giant selectivity and 100 nm minimal size, by means of wet etching with hydrofluoric acid. We therefore expect the observations here reported to be extensive to sapphire, with the exception that in sapphire or other anisotropic crystals optical writing is highly sensitive to crystallographic axis.

In conclusion, we report on a significant advancement on 3D laser nanolithography for nanophotonics, with a technique capable of inscribing both positive step-index changes and high contrast (air) structures, in both cases with 100 nm minimum feature sizes so far, and with high fidelity (∼10 nm) given by the 3D nanopositioning stages and the easiness to overlap photomodified regions in absence of damage or distortions. Results presented indicate that a new range of nanophotonic structures may be feasible with this disruptive technique, such as nanogratings and metaoptics, all embedded within hard YAG crystals. Due to the embedded, seamless, and monolithic nature of this technology and since YAG crystals can sustain temperatures of up to 1900 °C and are highly chemically inert and erosion resistant, we also foresee its direct application in harsh real-world environments, potentially beyond what current lithographically-made nanostructured devices can withstand.
